# Kynurenic Acid Protects against Thioacetamide-Induced Liver Injury in Rats

**DOI:** 10.1155/2018/1270483

**Published:** 2018-09-20

**Authors:** Sebastian Marciniak, Artur Wnorowski, Katarzyna Smolińska, Beata Walczyna, Waldemar Turski, Tomasz Kocki, Piotr Paluszkiewicz, Jolanta Parada-Turska

**Affiliations:** ^1^Department of Pharmacology, Medical University, Chodźki 4a Street, 20-093 Lublin, Poland; ^2^Department of Biopharmacy, Medical University, Chodźki 4a Street, 23-093 Lublin, Poland; ^3^Department of Surgery and Surgical Nursing, Medical University, Szkolna 18 Street, 20-124 Lublin, Poland; ^4^Department of Clinical Pathomorphology, Medical University, Jaczewskiego 8b Street, 20-090 Lublin, Poland; ^5^Department of Experimental and Clinical Pharmacology, Medical University, Jaczewskiego 8b Street, 20-090 Lublin, Poland; ^6^Department of Rheumatology and Connective Tissue Diseases, Medical University, Jaczewskiego 8 Street, 20-090 Lublin, Poland

## Abstract

Acute liver failure (ALF) is a life-threatening disorder of liver function. Kynurenic acid (KYNA), a tryptophan metabolite formed along the kynurenine metabolic pathway, possesses anti-inflammatory and antioxidant properties. Its presence in food and its potential role in the digestive system was recently reported. The aim of this study was to define the effect of KYNA on liver failure. The Wistar rat model of thioacetamide-induced liver injury was used. Morphological and biochemical analyses as well as the measurement of KYNA content in liver and hepatoprotective herbal remedies were conducted. The significant attenuation of morphological disturbances and aspartate and alanine transaminase activities, decrease of myeloperoxidase and tumor necrosis factor-*α*, and elevation of interleukin-10 levels indicating the protective effect of KYNA in thioacetamide (TAA) - induced liver injury were discovered. In conclusion, the hepatoprotective role of KYNA in an animal model of liver failure was documented and the use of KYNA in the treatment of ALF was suggested.

## 1. Introduction

Acute liver failure (ALF), the essence of which lies in acute massive hepatocyte necrosis, may lead to shock, coagulation disorders, encephalopathies, brain oedema, renal failure, infection, and development of multiple organ failures [1]. The most common causes of acute liver failure are drug-induced liver damage [2], viral infection [3], and autoimmune diseases [4]. Treatment of ALF is based on adjunctive therapy carried out until the proper functioning of the liver is reestablished. The definitive treatment of acute liver failure is the emergency liver transplant. In selected patients, for whom donors are unavailable, the support with bioartificial liver and complex critical care protocols are considered as lifesaving bridge therapy. In some cases, the bridge measures lead to survival when the liver function spontaneously recovers [5, 6]. The mechanism of spontaneous restoration of liver function after acute failure is under debate [7]. Therefore, there is a continuing need for other treatment options and protective measures decreasing the risk of liver failure.

Micro ingredients of food can play an important role in the regulation of many physiological and pathological pathways. Kynurenic acid (KYNA), a tryptophan metabolite formed along the kynurenine metabolic pathway is present in some food products in various concentrations. The so-called “Mediterranean diet” is exclusively rich in KYNA. KYNA is an antagonist of the ionotropic glutamate receptors [8] and *α*7 nicotinic receptor [9]. Moreover, it is an agonist of the G protein-coupled receptor (GPR35) [10] and aryl hydrocarbon receptor (AHR) [11]. Its neuroprotective, anticonvulsant, anti-inflammatory, and antioxidant qualities are thoroughly documented [12, 13]. The importance of KYNA in the digestive system is highlighted by several reports, however, its role in hepatic function is not elucidated. KYNA was found to be present in bile and intestinal mucus [14] in high concentrations enabling its interaction with receptors mentioned above. KYNA was found to protect against gastroduodenal ulceration in mice and rats [15, 16] and to inhibit intestinal hypermotility and inflammatory activation in experimental colon obstruction in dogs [17] and experimental colitis in rats [18]. Moreover, the content of KYNA is decreased in humans with irritable bowel syndrome [19, 20].

The aim of this study was to examine the effect of KYNA in the rat mode of acute liver injury induced by thioacetamide (TAA).

## 2. Material and Methods

### 2.1. Animals

Adult male Wistar rats were purchased from the Laboratory Animal Breeding Centre (Zbigniew Lipiec, Brwinów, Poland). Animals were housed in standard conditions (temperature of 21°C, 50–55% relative humidity, and 12 h light/dark cycle) with free access to food and water. After arrival, rats were acclimatized for seven days before use.

### 2.2. Drugs and Reagents

KYNA and TAA were obtained from Sigma-Aldrich (St. Louis, MO, USA). KYNA was dissolved in water followed by the addition of 1 N NaOH and then diluted to the final concentration of 100 mg/mL. TAA was dissolved in a saline solution to the final concentration of 30 mg/mL. Thiopental was obtained from Biochemie (Austria) and diluted with a saline solution to a final concentration of 50 mg/mL.

Hepatoprotective herbal remedies in the form of tablets were purchased from commercial shops. The list of used herbal drugs and their producers is presented in Table 1.

All high-performance liquid chromatography (HPLC) reagents were purchased from J.T. Baker (Deventer, Netherlands) and were of the highest available purity: zinc acetate (analytical purity 100%), sodium acetate (analytical purity 100%, assay by nonaqueous titration), water (HPLC gradient grade), acetonitrile (HPLC ultra gradient grade), and isopropyl alcohol (99.5%). Hydrochloric acid (J.T. Baker; Deventer), sodium hydroxide, and trichloroacetic acid (both POCH, Gliwice, Poland), which were used to prepare standards and samples, were of analytical grade. For extraction of KYNA, the cation exchange resin Dowex 50WX4-400, purchased from Sigma-Aldrich, was used.

### 2.3. Ethics Statement and Experimental Design

The experiments were performed according to the rules of animal care and were approved by the First Local Ethics Committee for Animal Experimentation in Lublin, Poland (resolution number 37/2011). The experiments were carried out using eight- to ten-week-old male Wistar rats weighing 277.5 ± 5.66 g. All efforts were made to minimize animal suffering and to reduce the number of animals used. Rats were randomly divided into four groups (*n* = 8 per group): (1) control group: control animals treated with saline solution; (2) KYNA group: treated with KYNA intraperitoneally (ip.) at a dose of 500 mg/kg body weight (b.w.) and after 30 minutes with a saline solution; (3) TAA group: treated with TAA ip. (150 mg/kg b.w.) and a saline solution ip. 30 minutes before TAA; (4) KYNA + TAA group: treated with KYNA ip. (500 mg/kg b.w.) and after 30 minutes with TAA ip. (150 mg/kg b.w.). 24 h after the TAA injection, animals were anesthetized with thiopental (50 mg/kg b.w.). No animals died during this period. Inferior caval vein blood was collected for plasma and serum, then the samples were centrifuged at 4000 rpm for 15 minutes. The serum and plasma were stored at −80°C for further analysis. The liver was immediately excised and cut into two specimens. One was fixed in 4% paraformaldehyde for histological examinations, and the other was frozen in liquid nitrogen and then stored at −80°C until further analysis.

### 2.4. Liver Function Test—Activity of Serum Transaminases

The activities of alanine transaminase (ALT) and aspartate transaminase (AST) were measured in all groups using the Siemens Dimension RxL chemistry analyzer with Alanine Aminotransferase and Aspartate Aminotransferase Flex reagent cartridges (Siemens Healthcare Diagnostics Inc., Newark, USA) according to the manufacturer's instruction.

### 2.5. Histological Examination

The paraformaldehyde-fixed liver tissues of each group were processed by routine histological procedures, embedded in paraffin, and 5 *μ*m sections were cut from the blocks. The paraffin-embedded liver sections were stained with haematoxylin-eosin for histopathological examination. Each sample was examined in a blinded manner with light microscopy (Olympus Manual System Microscope BX43) and observed at 20x and 40x magnification.

### 2.6. Concentration of Thiol Groups (–SH) in Tissue Homogenates

Liver samples were homogenized with cold Tris-HCl buffer (pH 7.2) and centrifuged at 10,000 rpm for 15 minutes at 4°C. The supernatant was used to assay the level of oxidative protein damage, based on the measurement of the concentration of thiol groups (–SH) in liver homogenates, using the Ellman reaction (DTNB reagent) and photometry at 405 nm (PHERAstar FS spectrometer—BMG Labtech GmbH, Germany).

### 2.7. Assessment of Oxidative Stress

Liver samples were homogenized with cold phosphate buffer (pH 7.4) and centrifuged at 4000 rpm for 30 min at 4°C. The supernatant was used to assay malondialdehyde (MDA) and 4-hydroxynonenal (4-HNE) levels, myeloperoxidase (MPO) activity, and heme oxygenase 1 (HO-1) concentration. The levels of MDA and 4-HNE were measured using the MDA + 4HNE assay kit (OxisResearch Products Percipio Biosciences, Oregon, USA), MPO activity was measured using the MPO assay kit (Hycult Biotech, Pennsylvania, USA), and HO-1 concentration was measured using the HO-1 assay kit (Stressgen, Pennsylvania, USA), following the manufacturer's instructions and using photometry at 586 nm (MDA + 4HNE) or 450 nm (MPO and HO-1) (PHERAstar FS spectrometer). Oxygen radical absorbance capacity (ORAC) was quantified on the basis of the decrease in the fluorescence of the so-called molecular probe, fluorescein. Inhibition of oxidative damage to the fluorescent probe can be correlated with the antioxidant capacity of a compound acting as a free radical scavenger. The ORAC assay was performed as described by Huang et al. [21] using ORAC Antioxidant Assay Kit (Zenbio, North Carolina, USA) and using fluorescence spectroscopy at 485 and 528 nm (PHERAstar FS spectrometer).

### 2.8. Determination of Pro- and Anti-Inflammatory Cytokines

The levels of tumor necrosis factor-*α* (TNF-*α*) and interleukin-10 (IL-10) in plasma were measured by ELISA kits (R&D Systems, Minneapolis, MN, USA) according to the manufacturer's instructions. Absorbance measurements were carried out at 450 nm using PHERAstar FS spectrometer.

### 2.9. Determination of KYNA

Rat liver samples were homogenized in distilled water (1 : 5 w/v) and centrifuged (12,000 rpm, 20 min). The supernatant was collected for KYNA determination. Distilled water was added to herbal tables (1 : 5 w/v). After 30 min they were homogenized, centrifuged (4000 rpm, 5 min), and the supernatant was collected for KYNA determination. KYNA was isolated and determined according to the methods described previously [22, 23]. Samples were applied to the columns containing cation exchange resin Dowex 50 and the fraction containing KYNA was eluted. The eluate was subjected to high-performance liquid chromatography (HPLC) and KYNA was detected fluorometrically (Hewlett-Packard, Palo Alto, CA, USA; 1050 HPLC system: ESA catecholamine HR-80, 3 *μ*m, C18 reverse-phase column, mobile phase: 250 mm zinc acetate, 25 mm sodium acetate, 5% acetonitrile, pH 6.2, and flow rate of 1.0 mL/min; fluorescence detector: excitation 244 nm, emission 398 nm).

### 2.10. Determination of Proteins

Sample protein contents were estimated by Bradford's method using bovine serum albumin as a standard [24].

### 2.11. Bioinformatic Analysis of Gene Expression

Data on gene expression were extracted from Gene Expression Omnibus (GEO) and ArrayExpress repositories using Genevestigator v5.11.05 (Nebion AG, Zurich, Switzerland) [25]. Only samples from control (i.e., vehicle treated), wild-type animals were employed for rat gene expression profiling. Data from normal liver tissues of healthy individuals were used for analysis of human genes. Retrieved data were from Affymetrix Rat Genome 230 2.0 Arrays and Affymetrix Human Genome U133 Plus 2.0 Arrays. Extracted values were plotted as log_2_ using Prism 6 (GraphPad Software, La Jolla, CA, USA).

### 2.12. Statistical Analysis

The data were expressed as a mean value ± standard error of the mean (SEM) and analyzed using Statistica 12 software (Statistica Poland). Statistical significance among the groups was analyzed using the one-way analysis of variance ANOVA test with Tukey's post hoc test. *P* < 0.05 was considered statistically significant.

## 3. Results

### 3.1. KYNA Content in the Liver

The average content of endogenous KYNA in the liver homogenates in the control group was 62.6 ± 2.9 pmol/g. TAA administration decreased the endogenous KYNA concentration to 40.5 ± 2.8 pmol/g. Administration of KYNA (500 mg/kg, ip.) resulted in an increase of KYNA in the liver tissue to 239.9 ± 24.3 pmol/g and fully prevented the effect evoked by TAA (249.5 ± 10.1 pmol/g) (Table 2).

### 3.2. Effect of KYNA on Serum Aspartate and Alanine Transaminase Activities

AST and ALT activities measured in serum of control animals was 109.8 ± 7.2 U/L and 54.7 ± 4.6 U/L, respectively. KYNA affected neither AST nor ALT activity (145.3 ± 34.1 U/L; ALT 57.7 ± 5.2 U/L, resp.). TAA administration resulted in a marked increase in the levels of AST and ALT to 316.3 ± 59.3 U/L and 90.2 ± 12.9 U/L, respectively. The increase of AST and ALT activity induced by TAA was abolished by KYNA administration and came down to 185.6 ± 17.5 U/L and 57.8 ± 7.8 U/L, respectively (Table 2). AST/ALT ratio calculated in control animals was 2.0. KYNA infusion slightly elevated the AST/ALT ratio to 2.2. The significant increase of the AST/ALT value to 3.8 was observed after the administration of TAA and this ratio did not change significantly in the TAA group pretreatment by KYNA (Table 2).

### 3.3. Effect of KYNA on –SH Group Content in Liver

The content of –SH groups measured in liver homogenates of control animals (128.8 ± 27.0 mmol/mg protein) and KYNA-treated rats (108.3 ± 22.0 mmol/mg protein) did not differ significantly. TAA administration resulted in a decrease in the levels of –SH groups to 36.3 ± 10.0 mmol/mg protein. This decrease was partly attenuated by KYNA administration (61.5 ± 9.0 mmol/mg protein) (Table 2).

### 3.4. Effect of KYNA on Pro- and Anti-Inflammatory Cytokines

The content of IL-10 and TNF-*α* in the serum of rats in the control group was 421.1 ± 43.9 pg/mL and 22.2 ± 1.7 pg/mL, respectively (Table 2). KYNA administration raised IL-10 content (975.6 ± 141.9 pg/mL), but did not influence TNF-*α* level (19.4 ± 1.1 pg/mL) (Table 2). Administration of TAA did not affect IL-10 level (576.8 ± 54.5 pg/mL); however, it increased TNF-*α* production (39.1 ± 5.9 pg/mL) (Table 2). In rats treated with KYNA and TAA, the IL-10 content (909.2 ± 49.5 pg/mL) was higher in comparison with the control group (Table 2). The increase of TNF-*α* content induced by TAA was abolished by KYNA administration (27.1 ± 2.1 pg/mL) (Table 2).

### 3.5. Effect of KYNA on Oxidative Stress

TAA administration resulted in an increase in both MDA + 4HNE and MPO levels in the liver. These changes were abolished by KYNA pretreatment. KYNA alone affected neither MDA + 4HNE nor MPO content (Table 2). HO-1 content was raised above control level in all treatment groups (Table 2). ORAC value was decreased in the TAA-treated group. This effect was fully prevented by KYNA pretreatment (Table 2).

### 3.6. Effect of KYNA on Liver Morphology

**Figure 1:**
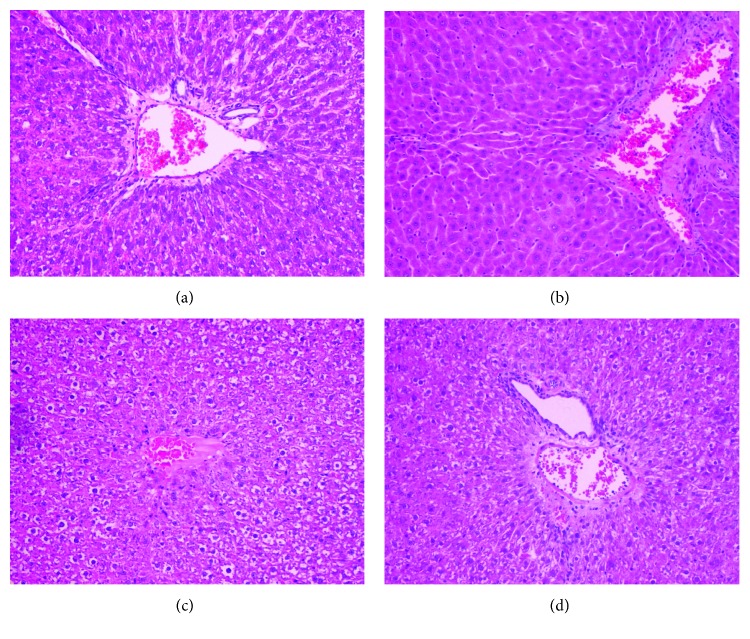
Effect of kynurenic acid (KYNA) on liver histology in thioacetamide (TAA) induced liver injury in rats. (a) Control group: normal cell structure and lobular architecture. (b) KYNA (500 mg/kg) treated group: normal cell structure and lobular architecture. (c) TAA (150 mg/kg) treated group: significant hepatocellular damage with severe inflammatory cell infiltration. (d) KYNA (500 mg/kg) and TAA (150 mg/kg) treated group: mild inflammation. Hematoxylin and eosin staining, magnification 40x.

Liver sections from the control and KYNA-treated animals presented a normal lobular architecture with radiating hepatic cords and clear central veins, with no trace of inflammation or necrosis (Figures 1(a) and 1(b)). Administration of TAA caused an occurrence of hepatocytes with multiple, large nuclei, parenchymal cell loss with limited portal stagnation, and inflammatory lobular changes (Figure 1(c)). KYNA injection 30 minutes before TAA significantly limited the extent of liver damage. KYNA pretreatment reduced interstitial cellular infiltration and stagnation in the portal tract space; it also limited the necrosis (Figure 1(d)).

### 3.7. Expression of KYNA-Related Genes in Rat and Human Liver Tissue

**Figure 2:**
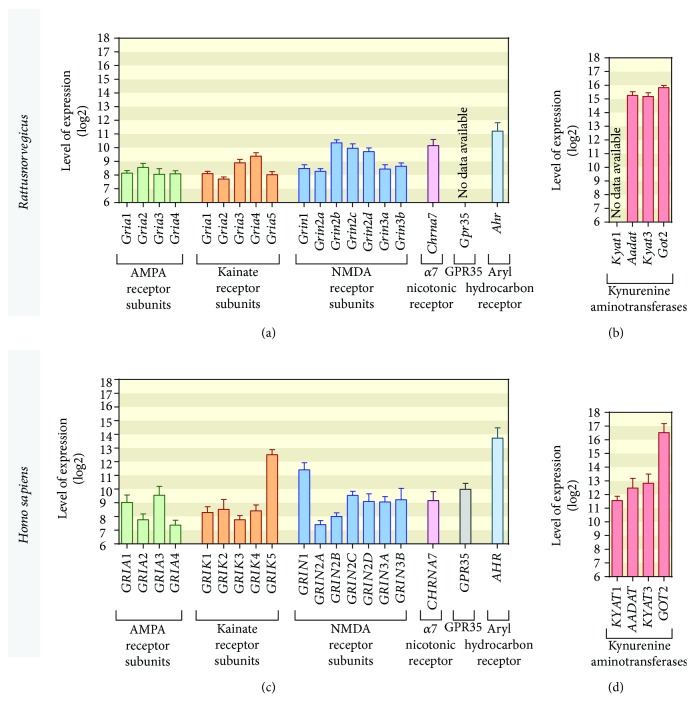
Expression of KYNA-related genes in rat and human liver tissue. (a) Expression of genes coding for *α*-amino-3-hydroxy-5-methyl-4-isoxazolepropionic acid (AMPA) receptor subunits (*Gria1*, *Gria2*, *Gria3*, and *Gria4*), kainate receptor subunits (*Grik1*, *Grik2*, *Grik3*, *Grik4*, and *Grik5*), N-methyl-D-aspartate (NMDA) receptor subunits (*Grin1*, *Grin2a*, *Grin2b*, *Grin2c*, *Grin2d*, *Grin3a*, and *Grin3b*), *α*7 nicotinic acetylcholine receptor (*Chrna7*), and aryl hydrocarbon receptor (*Ahr*) in rat liver tissue (*n* = 3502). There was no available data on G protein-coupled receptor 35 (*Gpr35*) expression. (b) Expression of *Aadat*, *Kyat3*, and *Got2* genes coding for kynurenine aminotransferases (KATs) in rat liver tissue (*n* = 3502). No probes were available for *Kyat1* gene within the selected array. Data on rat gene expression were retrieved from previously described [26–37] microarray experiments deposited in GEO (accession numbers: GSE5350, GSE5509, GSE8880, GSE10408, GSE11097, GSE11097, GSE10411, GSE10015, GSE12196, GSE14712, GSE57822, GSE73500) and ArrayExpress (accession numbers: E-MTAB-799, E-MTAB-800) databases. (c) Expression profile of human orthologues of the rat genes coding for KYNA-related receptors (*n* = 70). (d) Expression of human orthologues of KAT-coding rat genes (*n* = 70). Data on expression of the genes of interest in human liver tissues were extracted from previously described [38–44] microarray-profiling experiments available at the GEO repository (accession numbers: GSE23343, GSE11190, GSE3526, GSE37031, GSE55092, GSE28619, GSE7307, GSE43346).

In order to determine whether KYNA can exhibit its liver-protective activities directly at hepatocytes, we reexamined the expression profiles of *α*-amino-3-hydroxy-5-methyl-4-isoxazolepropionic acid (AMPA), kainate, N-methyl-D-aspartate (NMDA) ionotropic glutamate receptors followed by the *α*7 nicotinic receptor, GPR35, and AHR in previously published microarray experiments (Figures 2(a) and 2(c)). The analysis revealed that these KYNA-sensitive receptors are expressed in rat liver cells, although at varying levels (Figure 2(a)). No data on the expression of *Gpr55* were available on the selected microarray platform. Moreover, rat hepatocytes were found to express relatively high levels of *Aadat*, *Kyat3*, and *Got2* mRNA coding for three different kynurenine aminotransferase (KAT) isoforms (Figure 2(b)). There was no probe for *Kyat1* on the array of interest. Human hepatocytes displayed a similar expression pattern of KYNA-related genes (Figures 2(c) and 2(d)). Noteworthy, human liver cells were positive for both *GPR35* and *KYAT1* mRNA.

### 3.8. Content of KYNA in Herbal Remedies

KYNA presence was found in all sixteen tested hepatoprotective herbal remedies. The highest amount of KYNA was found in Hepato Protect—4.59 *μ*g/tablet, Cynarex—2.34 *μ*g/tablet, and Sylicinar—1.05 *μ*g/tablet. The lowest content of KYNA was recorded in Essetreen Complex—0.02 *μ*g/tablet and Syliflex—0.05 *μ*g/tablet (Table 1).

The amount of KYNA in a maximum daily dose of hepatoprotective herbal remedies was estimated according to the dosing recommendations specified by the supplier. The highest amount of KYNA in a daily dose is offered by Cynarex—14 *μ*g/day, Hepato Protect—9.2 *μ*g/day, and Sylicinar—6.3 *μ*g/day (Table 1).

## 4. Discussion

Liver failure induced by TAA mimics fulminant hepatitis in a clinically relevant manner. In rats, administration of TAA results in hepatic dysfunction, abnormal liver biochemical markers, and encephalopathy; all these features are hallmarks of ALF [26, 27]. Detailed molecular mechanisms of TAA-induced ALF are not well established [27]. However, it is known that the biotransformation of TAA to thioacetamide sulfoxide (TAAS) and subsequent conversion to its dioxide (TAAD) involves processing by CYP2E1. The second step for forming TAAD is less efficient than the first one. Finally, the two-step bioactivation of TAA-induced liver injury occurs in the initial time points ranging from 6 to 12 hours after TAA injection [28].

Covalent binding of TAA metabolites to liver macromolecules and increased reactive oxygen species production follows this process [29]. TAA induces both oxidative stress and lipid peroxidation [30]. It also causes a decrease in antioxidant potential [31]. In this study, TAA was administered ip. in a single dose of 150 mg/kg. This procedure resulted in an elevation of ALT and AST activity, an increase in oxidative stress, and an increase in the level of proinflammatory cytokines. Moreover, it lowered the level of thiol groups. Morphologically, the centrilobular liver necrosis was observed after TAA administration. Previous studies reported that the pathological effects produced by TAA are restricted to liver tissue [32]. Moreover, the reproducibility, relatively low cost, and investigator safety make TAA injection the most recommended model of ALF [27]. Thus, it was employed in our study.

KYNA is a metabolite of tryptophan formed along the kynurenine pathway. In the liver, this pathway starts with tryptophan 2,3-dioxygenase, which is responsible for the degradation of more than 90% of peripheral tryptophan [33]. The first metabolite within the pathway is an N-formyl kynurenine, which is rapidly transformed to kynurenine. The latter can be converted to KYNA by kynurenine aminotransferases, EC 2.6.1.7 [34]. In humans, KATs are encoded by four genes: *KYAT1*, *AADAT*, *KYAT3*, and *GOT2*; orthologues of these genes were identified in rats—*Kyat1*, *Aadat*, *Kyat3*, and *Got2* [35]. The available expression-profiling data showed that genes encoding KATs are effectively transcribed in liver tissues of both rats and humans (although no data on *Kyat1* expression was available). This stays in line with previous experiments demonstrating high activity of the enzymes and efficient production of KYNA in rat liver tissue [36] as well as KYNA secretion to bile in humans [14]. KYNA can be delivered to the liver from external sources, for instance, *via* intraperitoneal injection, as demonstrated in this study. However, the expressions of enzymes involved in the production of KYNA in the liver indicate that KYNA can be produced on-site. Thus, it is tempting to speculate that it is possible to mimic liver-protecting actions of KYNA using either KYNA precursors (i.e., kynurenine or tryptophan) or positive modulators of kynurenine aminotransferases. This hypothesis stays in line with the observation that the intraperitoneal administration of tryptophan [33] or D-kynurenine [37] leads to a significant increase in KYNA levels in rodent liver.

Here, we report that the pretreatment of rats with KYNA protected the animals from ALF caused by TAA and mitigated the TAA-dependent abnormalities in liver morphology. Intraperitoneal injection of 500 mg/kg of KYNA elicited a 4-fold increase of KYNA level in the liver. The dose of KYNA infused intraperitoneally was used for the experimental elevation of KYNA concentration in liver tissue at the time of initial liver injury that was expected 6–12 hours after TAA injection. Direct intraperitoneal injection of KYNA and TAA dismisses digestive absorption and probably abolishes the role of the bowel and its chemical and microbiotic content in ALF induction. Interestingly, the TAA decreased the content of endogenous KYNA in liver tissue by almost 30%. This effect was completely abolished by the pretreatment with KYNA. These observations may suggest that a decrease in KYNA concentration should be considered as a factor involved in liver failure. According to data presented above, the elevation of KYNA concentration can prevent an injury of liver tissue. Additionally, it is probable that continuous intravenous infusion of a micromolar dose of KYNA elevating their concentration in liver tissue in the first 6 hours after TAA exposition may abolish or attenuate liver injury.

In our hands, KYNA pretreatment offered protection against TAA-induced liver injury. In KYNA-treated animals, TAA did not elevate ALT and AST, enzymes widely recognised as indicators of hepatic injury [38]. The AST/ALT ratios known as the De Ritis ratio [39] were not changed after administration of KYNA in both control and TAA groups. The similar ratio of De Ritis may indicate that KYNA pretreatment attenuates but does not change the mechanism of the hepatotoxicity of TAA. Correspondingly, morphological changes in the liver were markedly limited pointing to the protective action of KYNA.

The mechanism of KYNA-protective action in the rat model of ALF has not been previously investigated. KYNA is a ligand of several cell-surface receptors expressed at different body sites. Thus, it can exert different physiological actions depending on the landscape of available receptors. KYNA is a well-established antagonist of AMPA, kainate, and NMDA ionotropic glutamate receptors [40]. Moreover, the *α*7 nicotinic acetylcholine receptor can be inhibited by KYNA [9, 41]. Apart from that, KYNA acts as an agonist of GPR35 [10] and AHR [11]. Genes encoding all these physiological receptor targets of KYNA are expressed in human liver cells, although at varying levels. Similarly, the query of functional genomics repositories of rat genes in liver revealed the expression of all KYNA-related receptor-coding genes, apart from *Gpr35*, for which no probes were available within the selected microarray platform. Although the mRNA levels for KYNA receptors were relatively low, especially when compared to the expression of KATs, it is possible that, at least in part, the liver-protecting activity of KYNA was driven by its interaction with the cell-surface receptors and modulation of downstream signaling pathways. KYNA-sensitive receptors, including GPR35, were also expressed in human hepatocytes, thus suggesting that KYNA may display its hepatoprotective activity also in the context of human cells. KYNA was previously demonstrated to inhibit lipopolysaccharide-induced TNF-*α* production in peripheral blood mononuclear cells and in CD14^+^ monocytes in a GPR35-dependent fashion [10]. In the same study, KYNA was unable to modulate the TNF-*α* in the unstimulated cells [10]. Here, we also observed no effect of KYNA on TNF-*α* level at basal conditions. Activation of GPR35 reduced cAMP and calcium (Ca^2+^) levels in cells. Moreover, GPR35 activation by KYNA may inhibit phosphorylation of protein kinase B (ART), extracellular signal-regulated kinase (ERK), and p38 mitogen-activated protein kinase (p38); it may also elevate the *β*-catenin level [42]. All of these cellular responses decreased inflammatory transcription factors such as NF-*κ*B and AP1 and reduced induction of TNF-*α*, HMBG1 (high-mobility group box 1), IL4 (interleukin 4), *α*-Def (*α* defensin), and iNOS (inducible nitric oxide synthase). These mechanisms were reviewed recently [43]. However, the TAA-induced boost in TNF-*α* production was effectively offset by KYNA treatment. Based on the similarities in KYNA effects on induced TNF-*α* production, one may speculate that some of the liver-protective effects of KYNA are mediated by GPR35. Nevertheless, involvement of the individual receptor subtypes in KYNA-mediated liver protection and downstream signaling pathways that drive the actions of KYNA remain to be elucidated.

KYNA exhibits anti-inflammatory and antioxidative activities [17, 18, 44, 45]. Thus, such activities should also be considered as a mechanism implicated in its hepatoprotective action since TAA exerted its toxic activity upon biotransformation to thioacetamide disulfoxide, which dramatically enhances the production of reactive oxygen species leading to liver tissue injury [29, 46]. In fact, in our study the TAA administration produced an increase in MDA + 4HNE and MPO contents and a decrease of ORAC and thiol group levels. The levels of MDA + 4HNE, which are the products of lipoperoxidation, are known to be important oxidative stress indicators [47]. MPO activity is directly correlated with neutrophil accumulation in tissues [48]. The level of thiol groups serves as a marker of the protein damage. These organosulfur compounds play an important role in antioxidant protection [49]. Interestingly, KYNA administration fully prevented a TAA-induced increase in both MDA + 4HNE and MPO levels. Moreover, it prevented the decrease of ORAC and markedly reduced the decrease in thiol group content in liver tissue from TAA-treated animals. All these results further confirm the hepatoprotective activity of KYNA.

Interestingly, HO-1 is a defense protein able to reduce the oxidative stress and inhibit the activation of inflammatory mediators [50], which was enhanced in TAA-treated rats. The HO-1 content was elevated also by KYNA. In rats pretreated with KYNA and subsequently treated with TAA, the HO-1 level remained enhanced. Similarly, administration of KYNA produced an increase in anti-inflammatory cytokine IL-10 content in serum and this effect remained unaffected by TAA treatment. On the other hand, TAA enhanced proinflammatory cytokine TNF-*α* content in serum. Nonetheless, KYNA pretreatment attenuated this effect. The presented results suggest that KYNA induced stimulation of HO-1 content; inhibition of release of TNF-*α* and stimulation of IL-10 may all be the essential mechanisms of the anti-inflammatory and cytoprotective actions of KYNA in ALF.

Since it was found that KYNA might exert pronounced hepatoprotective action in an animal model of chemically induced liver failure, its use as a new prophylactic or therapeutic means for preventing ALF should be considered. It was shown that KYNA is present in medicinal plants and herbal remedies [51]. Therefore, we analyzed the content of KYNA in selected hepatoprotective herbal products and found out that KYNA was present in all sixteen investigated agents. However, its content was relatively low since in most of the tested products the amount of KYNA was less than 1 microgram per tablet. Taking into consideration that the recommended daily dose of herbal products is usually 2–6 tablets, the KYNA intake due to the use of such remedies is low. The highest recorded content of KYNA was in the range of 1–5 micrograms per tablet and the calculated highest maximal KYNA daily intake approximated 6–14 micrograms. It should be noted that the estimated daily excretion of KYNA in human urine reached 3–5 milligrams [52]. It can be concluded that hepatoprotective herbal products deliver less than 0.5% of the daily KYNA requirement.

Summing up, we found that KYNA may exert pronounced hepatoprotective action in an animal model of chemically induced liver failure. Thus, the use of KYNA as a new prophylactic or therapeutic means for the treatment of ALF should be considered. Moreover, since KYNA is a constituent of food present in a considerably high amount in selected products [53], the use of diet containing high KYNA or KYNA-enriched supplements seems to be reasonable, especially in subjects with high risk of ALF.

## Figures and Tables

**Table 1:** Content of kynurenic acid (KYNA) in hepatoprotective herbal medicines.

Brand and supplier	Herbal ingredients^∗^	Content of KYNA (*μ*g/tablet)	Maximum daily dose^∗^ (tablets/day)	KYNA content in a maximum daily dose (*μ*g/day)
Hepato Protect A-Z Medica, Gdańsk, Poland	*Cynara scolymus* L. 0.2 g; *Polygoni avicularis* 0.075 g	4.59	2	9.2
Cynarex Herbapol, Wrocław, Poland	*Cynarae herbae extractum siccum*—5 : 1 (extractant : ethanol)—0.25 g	2.34	6	14.0
Sylicinar Herbapol, Poznań, Poland	*Cynarae herba extractum siccum* DER3—1 : 7 (extractant : water)—0.14 g	1.05	6	6.3
Hepason Complex ASA, Głubczyce, Poland	*Cynara scolymus extractum* 0.3 g	0.89	2	1.8
Hepatil Trawienie Teva, Warszawa, Poland	*Cynara scolymus extractum* (cynarine 5%); *Mentha piperita*; *Silybum marianum extractum* (80% silymarin)	0.86	2	1.7
Trawienie Hasco-Lek, Wrocław, Poland	*Raphani sativi nigri radicis extractum* 0.2 g; *Cynarae herbae extractum* 0.035 g; *oleum menthae* 0.015 g	0.63	3	1.9
Verdin Complexx US Pharmacia, Wrocław, Poland	*Cynarae herbae extractum* 0.1 g; *Folium Rosmarini extractum* 0.125 mg; *Curcuma longa extractum* 0.02 g; *oleum menthae granulatum* 24% 0.00164 g	0.59	4	2.4
Silivit	*Silybi mariani fructus extractum siccum*—20–34 : 1 (extractant : ethanol 90%)—0.2143 g	0.33	2	0.7
Verdin Hepatixx US Pharmacia, Wrocław, Poland	*Taraxacum officinale extract* 0.1 g; *Curcumae longae rhizoma extract* 0.0268 g; *Oleum menthae granulatum* 24% 0.0009 g	0.32	2	0.6
Sylimaron 100 Olimp Laboratories, Dębica, Poland	*Silybi mariani fructus extractum* 0.125 g	0.23	3	0.7
Entedral Tactica, Kraków, Poland	*Chamomillae extractum siccum*	0.19	2	0.4
Rapacholin C Herbapol, Wrocław, Poland	*Cynae herbae extractum spissum* 0.047 g; *raphani sativi nigri radicis extractum siccum cum carbo activatus* (1 : 1) 0.15 g; *menthae piperitae oleum* 0.015 g	0.15	6	0.9
Esseliv Forte Aflofarm, Pabianice, Poland	Phospholipids from soy	0.07	6	0.4
Silimax Filofarm, Bydgoszcz, Poland	*Silibi mariani extractum siccum* (silymarin 0.07 g)	0.07	3	0.2
Syliflex Herbapol, Poznań, Polska	Silymarin-phospholipid complex	0.05	6	0.3
Essetreen Complex Tactica, Kraków, Poland	Phospholipids from soy; *Oleum Carthami*	0.02	2	0.04

Data represent a mean value. Samples were determined in duplicates. SEM varied from 0.6 to 2.2% of mean value. ^∗^As specified by the supplier.

**Table 2:** Effect of kynurenic acid (KYNA) on biochemical parameters in thioacetamide (TAA) induced liver injury in rats.

	Control	KYNA	TAA	KYNA + TAA
KYNA content (pmol/g)	62.6 ± 2.9	239.9 ± 24.3^∗^	40.5 ± 2.8^∗^	249.5 ± 10.1^∗#^
AST (U/L)	109.8 ± 7.2	145.3 ± 34.1	316.3 ± 59.3^∗^	185.6 ± 17.5^#^
ALT (U/L)	54.7 ± 4.6	57.7 ± 5.2	90.2 ± 12.9 ^∗^	57.8 ± 7.8^#^
AST/ALT ratio	2.0	2.5	3.5^∗^	3.2
–SH groups (mmol/mg protein)	128.8 ± 27.0	108.3 ± 22.0	36.3 ± 10.0^∗^	61.5 ± 9.0^∗#^
MDA + 4HNE (nmol/g)	3.6 ± 0.2	3.6 ± 0.1	4.1 ± 0.2^∗^	3.5 ± 0.1^#^
MPO (ng/mg protein)	25.4 ± 2.6	31.4 ± 2.3	70.2 ± 2.6^∗^	55.5 ± 4.1^#^
HO-1 (ng/g)	283.3 ± 11.0	328.9 ± 17.2^∗^	349.9 ± 12.2^∗^	394.8 ± 11.3^∗#^
ORAC (TE/mL)	49.0 ± 0.7	48.7 ± 1.1	45.5 ± 0.4^∗^	53.0 ± 1.2^#^
TNF-*α* (pg/mL)	22.2 ± 1.7	19.4 ± 1.1	39.1 ± 5.9^∗^	27.1 ± 2.1^#^
IL-10 (mg/mL)	421.1 ± 43.9	975.6 ± 141.9^∗^	576.8 ± 54.5	909.2 ± 49.5^∗#^

Data represent a mean value ± SEM of 6–8 independent experiments. Statistical analysis was performed using one-way ANOVA with Tukey's post hoc test; ^∗^*P* < 0.05—as compared with control group; ^#^*P* < 0.05—as compared with the TAA group. Control—saline-treated group; KYNA—kynurenic acid- (500 mg/kg) treated group; TAA—thioacetamide- (150 mg/kg) treated group; KYNA + TAA—kynurenic acid (500 mg/kg) and thioacetamide- (150 mg/kg) treated group; AST—aspartate transaminase; ALT—alanine transaminase; –SH groups—thiol groups; MDA + 4HNE—malondialdehyde (MDA) + 4-hydroxynonenal (4-HNE) content; MPO—myeloperoxidase activity; HO-1—heme oxygenase 1 content; ORAC—oxygen radical absorbance capacity; TNF-*α*—tumor necrosis factor-*α*; IL-10—interleukin-10.

## Abbreviations

AHR: Aryl hydrocarbon receptor
ALF: Acute liver failure
ALT: Alanine transaminase
AMPA: *α*-Amino-3-hydroxy-5-methyl-4-isoxazolepropionic
AST: Aspartate transaminase
b.w.: Body weight
GPR35: G protein-coupled receptor 35
4-HNE: 4-Hydroxynonenal
IL-10: Interleukin 10
ip.: Intraperitoneally
KAT: Kynurenine aminotransferase
KYNA: Kynurenic acid
MDA: Malondialdehyde
MPO: Myeloperoxidase
NMDA: N-Methyl-D-aspartate
ORAC: Oxygen radical absorbance capacity
TAA: Thioacetamide
TNF-*α*: Tumor necrosis factor-*α.*
